# Accelerating action to reduce anemia: Review of causes and risk factors and related data needs

**DOI:** 10.1111/nyas.14985

**Published:** 2023-03-29

**Authors:** Sonja Y. Hess, Aatekah Owais, Maria Elena D. Jefferds, Melissa F. Young, Andrew Cahill, Lisa M. Rogers

**Affiliations:** 1 Institute for Global Nutrition and Department of Nutrition, University of California Davis, Davis, California, USA; 2 Micronutrient Forum, Washington, DC, USA; 3 Centre for Global Child Health, Hospital for Sick Children on behalf of Exemplars in Global Health, Toronto, Ontario, Canada; 4 Nutrition Branch, Centers for Disease Control and Prevention, Atlanta, Georgia, USA; 5 Hubert Department of Global Health, Rollins School of Public Health, Emory University, Atlanta, Georgia, USA; 6 Gates Ventures on behalf of Exemplars in Global Health, Seattle, Washington, USA; 7 Department of Nutrition and Food Safety, World Health Organization, Geneva, Switzerland

**Keywords:** anemia, anemia of inflammation, iron deficiency anemia, non-nutritional anemia, nutritional anemia

## Abstract

Anemia is a major public health concern. Young children, menstruating adolescent girls and women, and pregnant women are among the most vulnerable. Anemia is the consequence of a wide range of causes, including biological, socioeconomic, and ecological risk factors. Primary causes include: iron deficiency; inherited red blood cell disorders; infections, such as soil-transmitted helminthiasis, schistosomiasis, and malaria; gynecological and obstetric conditions; and other chronic diseases that lead to blood loss, decreased erythropoiesis, or destruction of erythrocytes. The most vulnerable population groups in low- and middle-income countries are often at the greatest risk to suffer from several of these causes simultaneously as low socioeconomic status is linked with an increased risk of anemia through multiple pathways. Targeted and effective action is needed to prevent anemia. Understanding the causes and risk factors of anemia for different population subgroups within a country guides the design and implementation of effective strategies to prevent and treat anemia. A coordinated approach across various expert groups and programs could make the best use of existing data or could help to determine when newer and more relevant data may need to be collected, especially in countries with a high anemia burden and limited information on the etiology of anemia.

## INTRODUCTION

Anemia is defined as a hemoglobin concentration below an age-, sex,-and pregnancy-specific cutoff.^1^ For nonpregnant women, for example, a hemoglobin concentration of 110–119 g/L is defined as mild anemia, a concentration of 80–109 g/L as moderate anemia, and a hemoglobin concentration below 80 g/L is considered severe anemia. Overall, anemia continues to be a health condition of major public health concern, with young children, menstruating adolescent girls and women, and pregnant women among the most vulnerable population groups. Anemia is associated with poor cognitive and motor development outcomes in children, increased morbidity and mortality in women and children, poor birth outcomes, and decreased work productivity in adults.^2–8^ Globally, over half a billion women 15–49 years of age and 269 million children aged 6–59 months were estimated to be affected by anemia in 2019.^9^ The present paper aims to review known causes and risk factors of anemia and to provide reflections on the most pressing issues to be addressed in accelerating reductions in the prevalence and severity of anemia. The paper was prepared as an input paper in support of the *Comprehensive framework for integrated action on the prevention, diagnosis, and management of anemia* led by the World Health Organization (WHO).

Globally, 30% (95% uncertainty interval [UI] 27–33%) of nonpregnant women 15–49 years of age were estimated to be anemic in 2019, a prevalence level that has remained unchanged from the year 2000 (31% [95% UI 28–34%]).^10^ In contrast, the prevalence of anemia in pregnant women decreased from 41% (95% UI 39–43%) to 36% (95% UI 34–39%) during that same time period.^10^ However, the progress on anemia reduction is not sufficient to meet the global anemia target set by the World Health Assembly (i.e., 50% reduction of anemia among women of reproductive age for each country by 2025).^10,11^ Among children aged 6–59 months, the global prevalence of anemia was 40% (95% UI 36–44%) in 2019, down from 48% (95% UI 45–51%) in 2000, but unchanged since 2010 (40% [95% UI 36–44%]).^9,10^ In 2019, the highest burden of anemia was found in the WHO Regions of South-East Asia and Africa; the prevalence of anemia among women exceeded 50% in 10 countries and 70% among children in 11 countries.^10^ Of note, data suggest a shift toward mild anemia; while the prevalence of mild anemia globally and in most regions stagnated from 2000 to 2019, important progress was achieved as the prevalence of moderate and severe anemia declined in most populations and regions.^10^

**FIGURE 1:**
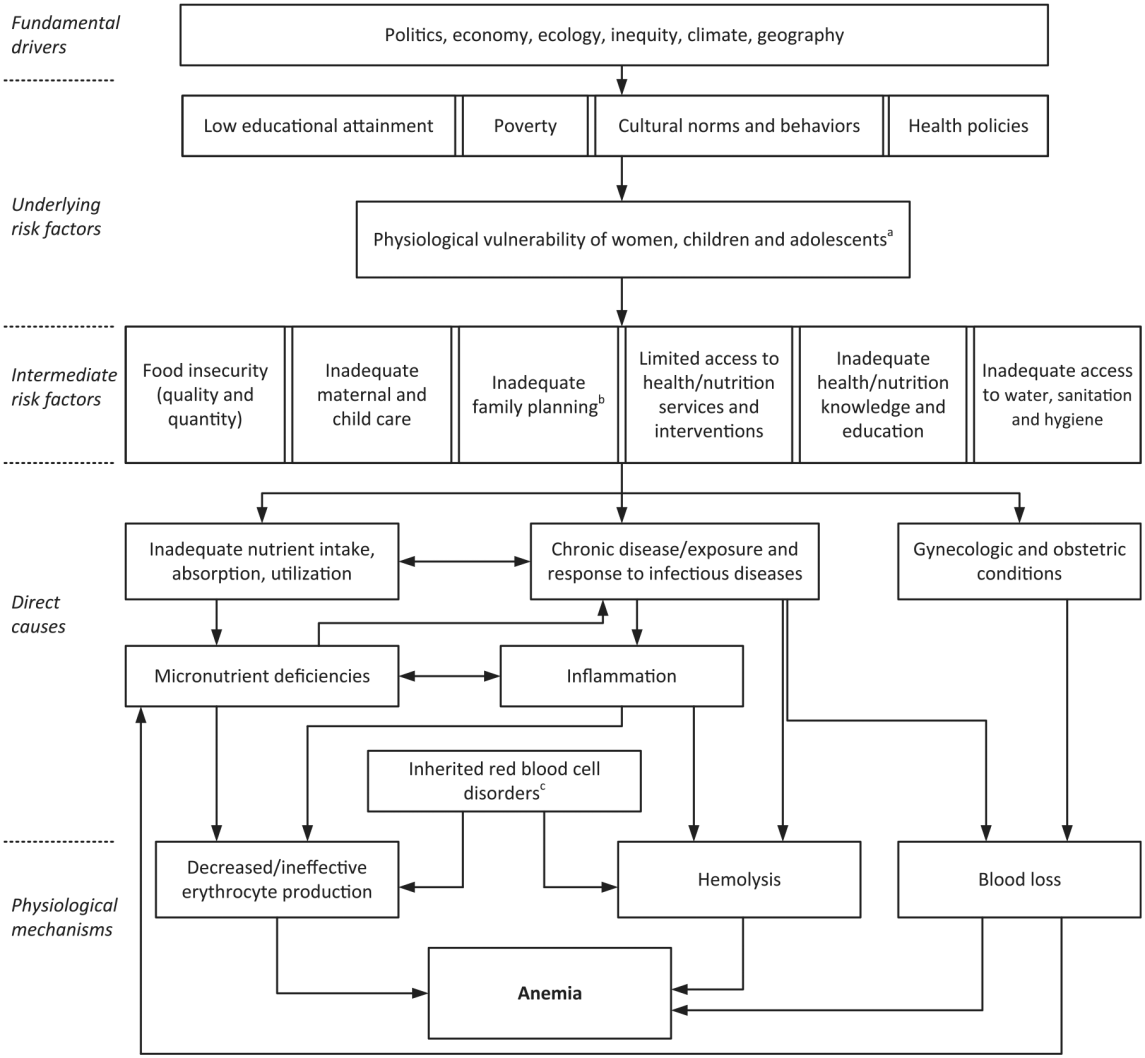
Conceptual framework of anemia etiology. Notes: ^a^High nutrient requirement due to growth, pregnancy, and increased susceptibility to infections during childhood and pregnancy. ^b^Early onset of childbearing, high parity, and short birth spacing. ^c^Inherited red blood cell disorders include sickle cell disorders, thalassemias, glucose-6-phosphate dehydrogenase (G6PD) deficiency, and other hemoglobinopathies. *Source*: Adapted and reproduced with permission from the publisher of reference.^6^ The original figure by Chaparro and Suchdev^6^ was developed based on determinants presented in references 53, 84, and 85.

Anemia is the consequence of a wide range of causes as well as biological, socioeconomic, and ecological risk factors,^6–8^ which often act concurrently (Figure 1). The three main underlying physiological mechanisms of anemia are: (1) ineffective erythropoiesis (i.e., inadequate production of erythrocytes); (2) hemolysis (i.e., erythrocytes are destroyed); and (3) blood loss.^6–8^

**FIGURE 2:**
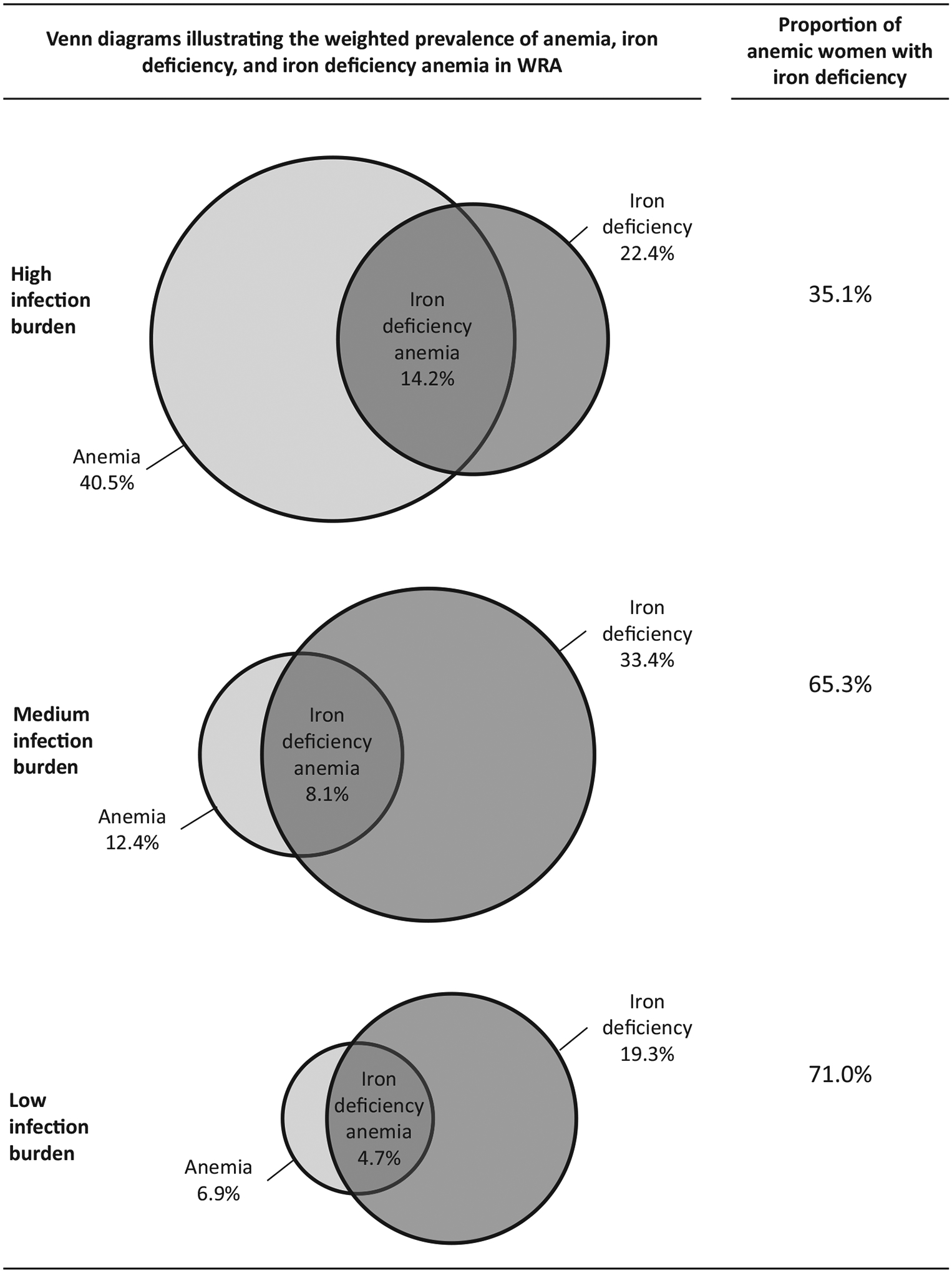
Venn diagrams illustrating the weighted prevalence of iron deficiency, anemia, iron-deficiency anemia, and the proportions of anemic women with iron deficiency in nonpregnant women of reproductive age by category of infection burden: results from the BRINDA project. Results from the BRINDA Project include nationally representative surveys. Iron deficiency was defined as an inflammation-adjusted ferritin concentration <15 μg/L. Anemia was defined as a hemoglobin concentration <120 g/L. Iron-deficiency anemia was defined as a hemoglobin concentration <120 g/L and inflammation-adjusted ferritin concentration <15 μg/L. Countries were categorized by infection burden as follows—low: Georgia and the United States; moderate: Colombia and Mexico (2006 and 2012); and high: Cameroon, Côte d’Ivoire, Liberia, and Laos. Abbreviation: WRA, women of reproductive age. *Source*: Wirth et al.^15^

Iron deficiency is considered the most common nutritional deficiency leading to anemia.^6^ Inadequate dietary iron intake is the primary pathway resulting in iron deficiency anemia. About 60% of the total global burden of anemia in 2019 was estimated to be due to dietary iron deficiency and thus iron deficiency accounted for the most significant cause of anemia-related disability.^12^ Deficiencies in vitamins A, B_2_, B_6_, B_12_, C, D, E, folate, copper, selenium, and zinc can also result in anemia due to their specific roles in the synthesis of hemoglobin and/or erythrocyte production;^6,7,13^ however, some of these micronutrients may not play a major role in the burden of anemia globally.^6,7^ Additional mechanisms include nutrient losses (e.g., iron deficiency secondary to blood loss from parasitic infections, hemorrhage associated with childbirth, or menstrual loss), impaired absorption (e.g., lack of intrinsic factor to facilitate vitamin B_12_ absorption or high intake of inhibitors, such as phytate that impair iron absorption), and nutrient interactions (e.g., vitamin A deficiency affecting mobilization of iron stores).^7^ Using data from nationally representative surveys, the project on Biomarkers Reflecting Inflammation and Nutritional Determinants of Anemia (BRINDA) recently assessed associations between anemia and multiple risk factors, such as iron and vitamin A deficiencies, inflammation, and malaria, and found that the proportion of anemic individuals who were iron deficient ranged from 30% to 71% in the different surveys and was negatively associated with the burden of infection/inflammation present in the population (assessed by elevated C-reactive protein and alpha-1-acid glycoprotein) (Figure 2).^14,15^

Inherited red blood cell disorders are other common causes of anemia, accounting for about 15% of the global burden of anemia in 2019.^12^ These include conditions such as: α- and β-thalassemia due to abnormalities of hemoglobin synthesis, sickle cell disorders due to changes in the hemoglobin structure, other hemoglobinopathies due to hemoglobin gene variants, abnormalities of red cell enzymes (glucose-6-phosphate dehydrogenase [G6PD] deficiency), or abnormalities of the red blood cell membrane (hereditary spherocytosis, elliptocytosis, and ovalocytosis).^16,17^ At least 5% of the world population carries a significant gene variant, but because of localized high carrier prevalence the highest prevalence of inherited red blood cell disorders is found in South-East Asia and Africa.^18^ This geographical localization of hemoglobinopathies is due to the partial protection against severe malaria. The proportion of anemia due to genetic hemoglobin and red blood cell disorders will likely continue to rise globally, particularly in low- and middle-income countries, as other causes, such as nutritional deficiencies and infectious diseases, are increasingly prevented.^19^ It is also likely to rise in higher-income countries due to population migration.

Anemia due to infections is another important cause of anemia globally, estimated to account for about 12% of total cases in 2019,^12^ and this is directly related to the geographical burden of infection. Infections can impair nutrient absorption and metabolism or can cause nutrient loss. Inflammation and chronic disease can also lead to anemia known as anemia of inflammation, which is immune-driven. Proinflammatory cytokines increase the synthesis of an iron-regulating hormone, hepcidin, which causes the sequestration of iron as ferritin, thereby blocking iron transfer to erythrocyte precursor cells in the bone marrow and resulting in decreased erythropoiesis as well as decreased erythrocyte survival.^20–23^ The exact pathological mechanisms depend on the underlying infection or disease causing the anemia.^23^ Soil-transmitted helminths, including hookworm (*Necator americanus* and *Ancylostoma duodenale*), *Trichuris trichiura*, and *Ascaris lumbricoides*, parasitize the gastrointestinal tract causing blood loss, which in turn can result in the loss of iron and the development of anemia.^24^ Globally, about 1.5 billion people are estimated to be infected with soil-transmitted helminths, with the highest infection rates in areas with poor sanitation in tropical and subtropical regions of sub-Saharan Africa, the Americas, China, and East Asia.^25^ Schistosomiasis is an infectious disease caused by trematode parasites of the genus *Schistosoma*. Adult worms live within the veins of the human host, mate, and cause anemia due to inflammation and blood loss.^26^ Other common infections that can cause anemia are *Helicobacter pylori* and visceral leishmaniasis.^27,28^ The primary causes of mild and moderate anemia tend to differ from those that cause severe anemia.^6^ Malaria is a major cause of anemia and is a primary cause of severe anemia.^6^ While malaria does not lead to iron loss, it alters iron metabolism through mechanisms that include hemolysis, release of heme, dyserythropoiesis, deposition of iron in macrophages, and inhibition of dietary iron absorption. Anemia is also very common among individuals infected with HIV and tuberculosis.^6,16^ HIV infection causes anemia through a wide range of mechanisms, including ineffective erythrocyte production, hemolysis, blood loss, and side effects of drug treatment. Chronic inflammation also causes anemia in tuberculosis patients.^16^

Various other conditions, such as gastrointestinal disease, kidney disorders, and other diseases, lead to blood loss, decreased erythropoiesis, or destruction of erythrocytes, resulting in anemia of inflammation, also referred to as anemia of chronic disease.^22^ This is considered the most frequent type of anemia among hospitalized and chronically ill patients. Globally, these diverse diseases are estimated to cause about 13% of anemia.^12,30^ Obesity is characterized by chronic mild inflammation, which has consistently been found to alter hematologic parameters and iron metabolism.^31,32^ However, even though obesity is associated with features of anemia of inflammation (i.e., alterations in iron homeostasis and hypoferremia),^22^ studies on associations between obesity and anemia have been inconsistent.^31,32^

Menstruating adolescent girls and women are among the most vulnerable population groups at risk of developing anemia because of the regular blood loss due to menstruation.^7^ This is aggravated during adolescence, a period known as the second growth spurt in life.^33^ During pregnancy, the risk of anemia increases due to the increased iron needs (e.g., for the placenta, the fetus, and expanded maternal blood volume during pregnancy) and potential blood loss during and after childbirth.^34^ Severe bleeding of postpartum hemorrhage is a risk factor for anemia.^35^ Every year about 14 million women primarily in low- and middle-income countries suffer from postpartum hemorrhage.^36,37^ In addition, pre-existing conditions, such as HIV, other infections, and noncommunicable diseases, may further exacerbate the risk of anemia during pregnancy.^38^

Importantly, the above-described causes can occur concurrently, and the most vulnerable population groups in low- and middle-income countries are often at the greatest risk to suffer from several of these causes simultaneously. Low socioeconomic status is linked with an increased risk of anemia via multiple pathways from poor living conditions, which include: poor water, sanitation, and hygiene; air pollution; smoking; food insecurity; and poor dietary quality (e.g., low dietary diversity predominantly relying on grain-based diets).^7,39^ Low attainment of formal education is another risk factor for anemia, as less formal education may affect a woman’s ability to access and understand the information provided on health, nutrition, family planning, and/or to earn a higher income, hence affecting nutritional security.^7^ A recent analysis of the national or subnational decline in anemia prevalence among women of reproductive age and the associated drivers in low- and middle-income countries found that healthcare utilization, especially seeking antenatal care during pregnancy and the use of contraceptives, were strong drivers for anemia reduction.^11^ Varying from setting to setting, there are health disparities by ethnicity and race,^7^ with minority groups often at increased risk of anemia likely due to the complex interplay of socioeconomic risk factors. Gender inequality, lack of women’s empowerment, and cultural practices related to early marriage and pregnancy can also contribute to the risk of anemia.^7,8^ In the above-mentioned, multicountry analyses of national and subnational anemia reduction, greater age at first pregnancy, higher body mass index, more birth spacing, and lower parity were associated with a modest reduction in anemia prevalence.^11^

## MAJOR GAPS IN KNOWLEDGE ON THE CAUSES AND RISK FACTORS OF ANEMIA AND WAYS FORWARD TO USE EXISTING DATA AND COLLECT NEW RELEVANT DATA

To prevent anemia among the most vulnerable population groups and accelerate progress toward the Global Nutrition Target 2 (50% reduction in the prevalence of anemia in women of reproductive age by the year 2025) and the Sustainable Development Goals (SDG) indicator 2.2.3 (prevalence of anemia in women 15–49 years of age, by pregnancy status),^40,41^ understanding the etiology of anemia is an important aspect in designing effective and targeted programs at the country or subnational level. In the following section, we describe current gaps and explore potential ways forward on improving the use of existing data and identifying data needed to better target anemia programs (Table 1). The points raised were derived from recent reviews of anemia, input from various experts from the WHO interdepartmental working group on anemia, STAGE (Strategic and Technical Advisory Group for Maternal, Newborn, Child, and Adolescent Health and Nutrition), and the Anemia Action Alliance. Approaches to improve the diagnosis of anemia and its causes, and to accelerate interventions and programs to prevent and treat anemia are reviewed elsewhere.^42–44^

WHO recently updated the national, regional, and global estimates of anemia for women 15–49 years of age (by pregnancy status) and children aged 6–59 months for the time period 2000–2019.^10^ Estimates for these population groups from 197 countries were based on 489 data sources collected in 133 countries spanning 1995–2020.^10,45^ Thus, hemoglobin data from population-representative surveys were lacking for 64 countries. The authors^45^ state that anemia estimates and trends over time were more reliable when countries had several data sources collected throughout this timeframe. At least three data sources were available for women in 64 countries (covering 72% of women globally) and for children in 63 countries (covering 60% of children globally); however, there were variations by region (Table 2).^45^ These recently updated global and regional estimates are similar to previous estimates despite updates to data sources and covariates.^46^ Moreover, anemia prevalence estimates produced for the Global Burden of Disease (GBD) Study 2019 are comparable for women of reproductive age despite differences in inclusion and exclusion criteria, statistical models, and covariates.^47^ The GBD estimates for children 0–59 months of age are slightly higher than those by Stevens et al. (46% [95% UI 44–48%] vs. 40% [95% UI 36–44%]), and not explained by the difference in the age range alone.^10,47^ Nevertheless, despite considerable data gaps and related uncertainty in the estimates for countries without recent nationally representative hemoglobin results, these estimates highlight that anemia continues to be a public health problem in many countries. Moreover, anemia prevalence estimates help determine whether the risk of anemia is severe, moderate, or mild in a country and can inform decisions regarding effective strategies to prevent and treat anemia, as well as opportunities to further assess and monitor the anemia situation in representative surveys.

WHO presently provides global anemia estimates only for women 15–49 years of age, by pregnancy status, and for children aged 6–59 months^9^ because these are the most vulnerable population groups for anemia. Data on hemoglobin concentration are even more limited for other population groups. Specifically, out of 432 nationally representative surveys carried out since 1990 and reported in WHO’s Micronutrients Database,^48^ hemoglobin was assessed primarily in the most vulnerable population groups, such as preschool-age children (68% of surveys), pregnant women (56% of surveys), and women of reproductive age (68% of surveys), with fewer surveys among other population groups, such as adolescents (53% of surveys; with about half of these including only older adolescents 15–19 years of age), men (27% of surveys), school-age children (23% of surveys), and older persons (11% of surveys). The decision on extending the survey to other population groups (such as adolescents or older persons) is guided by many factors and could include the assumed anemia risk, the prevalence of relevant causes among different population groups, and costs. Available resources can be a limiting factor in determining the number of indicators collected and the number of population groups assessed.

As described above, causes of anemia are both nutritional and non-nutritional, which are further aggravated by numerous socioeconomic and ecological risk factors caused by inequity (Figure 1). Thus, preventing anemia requires a coordinated, multisectoral, and strategic approach.^7^ Similarly, the collection and interpretation of data for understanding the causes and burden of anemia within a country or region can require a multidisciplinary approach. While there is extensive information available on malaria in children from sub-Saharan Africa, soil-transmitted helminths infections, and HIV/AIDS among adults,^25,49–51^ there is a sparsity of data on various other causes of anemia for different population groups. For example, there is less information on malaria among adults or HIV/AIDS among children. Also, there have been few population analyses of the genetic variants of different inherited red blood cell disorders.^16,17^ Due to this lack of information, it is likely that the prevalence of inherited red blood cell disorders and the respective global burden of anemia due to this cause is presently underestimated. The GBD 2019 study estimates the proportion of total anemia prevalence attributable to 35 causes based on cause-specific hemoglobin shifts, the estimated prevalence of known causes, and the overall hemoglobin distribution for each location, year, and population group from 1990 to the present.^47^ Because of the data sparsity, the GBD study does not use indicators of iron status to estimate the prevalence of iron deficiency, instead, it relies on counterfactual modeling and attempts to isolate the disease burden due to iron deficiency using hemoglobin as a proxy.^52^ Systematic data collection of various causes and risk factors of anemia from population-representative surveys could contribute to a better understanding of the complex interplay of causes and risk factors associated with the occurrence of anemia.

Despite the above-mentioned data gap, hemoglobin is among the most frequently assessed indicators in national nutrition surveys.^48^ However, national survey results are not always easily accessible in the public domain. With considerable efforts, the WHO’s Vitamin and Mineral Nutrition Information System (VMNIS) systematically retrieves and summarizes hemoglobin and other micronutrient status results from population-representative surveys.^48^ A public data repository of deidentified individual data that includes hemoglobin, as well as recommended indicators of causes and risk factors of anemia (Table 3), from population-representative surveys could facilitate and maximize the use of existing survey data and allow for standardized analyses to generate harmonized and comparable data among and within countries.

In recent years, data sharing of deidentified individual-level data has become more common and has resulted in important findings such as those from the BRINDA project,^14,15,53–55^ and the findings from the Exemplars in Stunting Reduction project.^56^ Sharing deidentified individual data across surveys, disciplines, and countries may allow for further exploration of available data in secondary analyses. Similarly, sharing properly stored biospecimens for analysis of additional biomarkers, such as indicators of micronutrient status or specific infections, could help elucidate causes and risk factors of anemia in various contexts. Importantly, before sharing and using samples and protected health information, investigators need to comply with relevant Institutional Review Boards and the privacy standards of the respective countries. Examples include the Health Insurance Portability and Accountability Act in the United States and the European Union General Data Protection Regulation.^57,58^ When data are exchanged or shared, developing material and data transfer agreements to define the conditions for data and specimen sharing between the data owners and researchers is highly recommended and has become a common practice.^59,60^

Because cross-sectional surveys do not allow causal attribution, data analyses across multiple data sources are recommended to determine the most likely underlying risk factors and causes of anemia.^61^ It can be challenging to identify various sources and bring all relevant sectors together for a comprehensive review of the data. Initiatives, such as the West African Health Organization (WAHO) and African Population and Health Research Center’s *Countdown to 2030* and the National Information Platforms for Nutrition (NiPN), are examples that provide support regionally, or to specific countries, to strengthen the management, in-country capacity, and use of nutrition data for effective national planning and decision-making.^62,63^ Such initiatives are potential resources to address the challenges with comprehensive data review for anemia prevention and reduction; however, not all regions or countries have access to these types of initiatives. Another challenge to interpreting anemia results is when two national surveys conducted close in time and assess the same population groups, especially young children, result in vastly different anemia prevalence estimates,^64^ leading to uncertainties regarding the level of the public health problem, actions warranted, and effectiveness of programs. More details on this topic are reviewed by Garcia-Casal et al.^42^ An additional consideration is the use of appropriate analytic methods to understand the etiology of anemia. For example, attributable fractions of anemia are sometimes used to examine etiology; however, there are multiple approaches and some are more complex than others, or even inappropriate, when anemia is not rare (e.g., Levin’s formula with odds ratio), which can result in potentially biased results.^65^ Furthermore, there are limitations to note when attributable fraction analyses are conducted using cross-sectional data because there may be reverse causality and it is not possible to draw causal inferences on true risk reduction of anemia.

While existing data can guide program designs and identify potential interventions, existing data can also be used to identify data gaps. If there are data gaps, it may be possible that administrative data or other proxy data could be leveraged to gain some insights into potential risk factors. Identifying data gaps is important to highlight the limits of our current understanding of the etiology of anemia and to guide future data collection.

In addition to leveraging existing data, there is a need to periodically collect new data. This is particularly important for countries with outdated or no data on anemia prevalence in high-risk populations. Countries that might most benefit from new data include those with a potentially high burden of anemia, limited data on the etiology of anemia, or those with recent changes in health programs or economic, social, or natural disasters. Planning and implementing a national survey is a major undertaking and requires a number of considerations and decisions, which are described in detail in the *Micronutrient survey manual*.^66^ Moreover, decisions regarding the inclusion of specific indicators may vary depending on the context and should be guided by the known or assumed prevalence of micronutrient deficiencies (including iron), burden of malaria and other parasitic infections, and prevalence of inherited red blood cell disorders (Table 3). An additional consideration for the choice of indicator(s) included in a survey is the availability of prevention or treatment strategies for the respective causes of anemia with the overarching goal that the survey results inform the design and implementation of new strategies aimed at addressing anemia or aid in the monitoring of existing programs. Survey results may also further guide which assessments could be prioritized in future population-representative surveys.

The inclusion of indicators on micronutrient status, particularly iron status, in the survey, depends on the objectives of the survey and whether the proportion of anemia due to iron deficiency is already known.^16^ There are several biomarkers of iron status that reflect different stages of iron sufficiency, and serum ferritin and soluble transferrin receptor are the most commonly used. WHO has recently updated guidance on the assessment of serum ferritin concentrations to include a recommendation to also measure markers of inflammation (e.g., C-reactive protein and/or α−1 acid glycoprotein) for the purpose of statistically correcting serum ferritin for inflammation.^67^ While deficiencies of some micronutrients (such as vitamins B_6_, C, D, E, copper, selenium, and zinc) may not contribute much to the global anemia burden, deficiencies in vitamins A, B_2_, B_12_, and folate could potentially be important in some contexts.^16,68–71^ Although there has been an increase in awareness of the importance of these micronutrient deficiencies due to their adverse health effects, there is a sparsity of information on deficiency prevalence^72^ and thus estimating the prevalence of various micronutrient deficiencies and their respective anemia-attributable burden remains challenging.^52^

In malaria-endemic countries, assessing malaria prevalence is warranted. Rapid diagnostic tests are inexpensive (cost approximately 1 US$) and are easy to administer.^66,73^ The assessment of indicators of parasitic infections depends on the burden of soil-transmitted helminth and schistosome infections.^74^ The inclusion and analytical approach to assess inherited red blood cell disorders can be informed by the type and geographic burden of inherited red blood cell disorders.^75–77^ Table 3 provides an overview of indicators of relevant causes and risk factors of anemia for consideration in surveys.

In countries with prevention programs targeting any of the major causes of anemia, such as iron supplementation or fortification, malaria prevention, deworming, or WASH (i.e., Water Access, Sanitation, and Hygiene), the reach, coverage, and quality of these programs should be assessed to determine the programs’ effectiveness.^7^ Monitoring and evaluation of programs allows for the identification and correction of any programmatic issues to improve program effectiveness. Malaria surveillance, for example, is considered a key aspect of intervention in countries that are malaria-endemic or have eliminated malaria but remain susceptible.^73,78^ It is important to note that even if a successfully implemented program preventing iron deficiency, malaria, or soil-transmitted helminthiasis and schistosomiasis, has only a minor impact on anemia, these programs are important for their own sake due to the substantial health burden of these causes per se.^24,34,49^

There are technical concerns related to hemoglobin assessment, such as the type of blood drawn (e.g., venous vs. capillary blood), the quality of the blood sample, and the analytical method and devices used for measuring hemoglobin, among other preanalytic, analytic, and postanalytic considerations.^79–82^ The gold standard for hemoglobin determination involves the processing of venous blood in an automated hematology analyzer and following appropriate quality control measures and standards,^79,80^ although the use of venous or pooled capillary blood samples in point-of-care devices with regular quality control against international reference standards could be considered as reviewed by Garcia-Casal et al.^42^

There are six global nutrition targets that have been endorsed by the World Health Assembly, five of which relate to children (stunting, wasting, overweight, obesity, and low birth weight) and the one target in women 15–49 years of age calls for a reduction in the prevalence of anemia by 2025.^40^ As 2025 nears, there will be an opportunity to revisit and revise these targets or call for their extension to 2030 to align with the United Nations’ SDGs.^41^ Although actions that effectively address conditions relating to the current six global nutrition targets may contribute to reducing childhood anemia, the addition of a global target on reducing anemia in children under 5 years of age could also be considered to draw needed attention and action to reducing the global burden of anemia,^83^ currently estimated at 269 million children.^9^ In the meantime, countries may decide to set their own national targets as they implement and monitor their programs designed to prevent and manage anemia.

In summary, causes of anemia span across multiple subject areas, including iron and other micronutrient deficiencies, various parasitic infections, inherited red blood cell disorders, gynecological and obstetric conditions, and various chronic diseases, which are further complicated by numerous interconnected socioeconomic and ecological risk factors. A coordinated and strategic approach across various expert groups and programs could make the best use of existing data, or could help to determine when newer and more relevant data may need to be collected among vulnerable population groups, especially in countries with a high anemia burden and limited information on the etiology of anemia with a focus on preventable or treatable causes of anemia. The complex interplay of causes and risk factors of anemia underscores the need for well-coordinated multisectoral action, including poverty reduction, women’s empowerment and equity, as well as improvements in nutrition and health. Such coordinated action may reduce not only the burden of anemia but also other health outcomes.

## Figures and Tables

**TABLE 1:** Major gaps in knowledge on the causes and risk factors of anemia and ways forward to use existing data and collect new relevant data.

Gaps in knowledge	Way forward
At present, 64 countries have no anemia information.	Consider hemoglobin data collection in nationally representative surveys in countries with outdated, little, or no available data.
Hemoglobin is assessed in national surveys primarily among the most vulnerable population groups (children and women of reproductive age).	Consider extending data collection to other population groups (such as older adolescents and older people), as resources permit.
Sparsity of data on various causes and risk factors of context-specific anemia.	For newly planned surveys, consider the inclusion of context-specific indicators (see Table 3).
Because of the complex interplay of nutritional and non-nutritional causes and risk factors of anemia, relevant data may come from a variety of sources.	Survey planning, collection of data, and interpretation of results require a coordinated, multidisciplinary approach.
Available data not always used to full potential.	Sharing of deidentified data and biospecimen and public repository of deidentified datasets may improve the use of existing data.
Technical concerns related to hemoglobin assessment.	Gold standard is the determination of hemoglobin in venous blood samples by automated hematology analyzer, although the use of venous or pooled capillary blood samples in point-of-care devices using quality control with international reference standards could be considered.^42^

**TABLE 2:** Number of surveys reporting anemia prevalence in children 6−59 months of age and women 15−49 years of age used in generating WHO global estimates of anemia 2000−2019, by WHO region.

WHO region (number of countries)	Americas (35)	African (47)	Eastern Mediterranean (22^a^)	European (53)	South-East Asia (11)	Western Pacific (27^b^)
Number of surveys	94	160	42	51	48	94
Average number of surveys per country	2.7	3.4	1.9	1.0	4.4	3.5
Number (%) of countries in region without survey data	10 (29%)	8 (17%)	6 (27%)	32 (60%)	0	6 (22%)

*Note*: Verified surveys reported in the WHO Micronutrients Database as of March 30, 2022.

Abbreviation: WHO, World Health Organization.

a Including Occupied Palestinian Territory.

b Taiwan, China counted as part of China.

**TABLE 3:** Indicators of relevant causes and risk factors of anemia to be considered for assessment in surveys, surveillance, or program monitoring.

Causes and risk factors of anemia	Diagnostic test, biomarker, or characteristics to identify condition	Proposed cutoff values or defining characteristics	Feasibility of collection	Cost
*Causes*				
Iron deficiency	Ferritin	Infants and children < 5 years (< 12 μg/L); children, adolescents, and adults 5 years and older (< 15 μg/L); pregnant women (< 15 μg/L)^67^	Often requires venous blood collection but a sandwich enzyme-linked immunosorbent assay technique enables the collection of pools of capillary blood; cold chain required	$-$$$
	C-reactive protein, alpha-1-acid glycoprotein	Inflammation defined as CRP > 5 mg/L or acid glycoprotein > 1 g/L	Biomarkers of inflammation are required for accurate interpretation of ferritin^67^	
Infections	Parasitic (malaria, soil-transmitted helminthiasis [STH], schistosomiasis [SCH])	Usually defined by the presence of infectious organism	Malaria and hematuria can be measured in the field from capillary blood and urine, respectively^73^	$-$$
	Viral (HIV/AIDS, hepatitis C, respiratory viruses including COVID-19)		Mapping of STH and SCH is based on stool (STH and SCH) and urine (SCH) specimens^74^	
	Bacterial (tuberculosis, salmonella, *H. pylori*, others)		Polymerase chain reaction testing diagnostic capacity variable for other infections	
Inherited red blood cell disorders	Abnormalities of hemoglobin synthesis (alpha or beta thalassemia)	Usually defined with molecular tests by genotyping DNA extracted from dried blood cards	May be collected using dried blood cards, so is not dependent on venous blood collection or cold chain	$-$$
	Abnormalities of hemoglobin structure (Hb S, C, and E)	Hemoglobin electrophoresis can also be used to detect Hb S, C, and thalassemia		
	Abnormalities of red cell enzymes (G6PD deficiency)	Genotyping or phenotyping tests can be used; rapid diagnostic test kits for qualitative phenotyping tests available (typically below 30–40% of normal activity)^76^		
	RBC membrane disorders (hereditary spherocytosis, elliptocytosis)	RBC membrane disorders can be diagnosed by RBC cytology, flow cytometry, ektacytometry, electrophoresis of RBC membrane proteins, and genetics^75^		
Blood loss	Onset of menstruation/menopause Hormonal contraception use Heavy menstruation Uterine fibroids Pregnancy and/or delivery complications	Usually defined by participant recall to questionnaires that include information about these characteristics	Requires the knowledge of cultural norms around reproductive health and birth	$
Deficiencies in other micronutrients	Vitamin A	Retinol < 0.7 μmol/L (although for women is still uncertain)^68^	Requires venous blood collection and cold chain, although folate can also be determined using dried blood cards. Limited availability of laboratories with externally validated performance	$$-$$$
	Riboflavin	Erythrocyte glutathione reductase activity coefficient > 1.3^69^		
	Folate	Serum folate < 6.8 nmol/L (risk of megaloblastic anemia) or RBC folate < 748 nmol/L (risk of neural tube defects)^a,70^		
	Vitamin B_12_	< 150 pmol/L (risk of megaloblastic anemia)^71^		
*Risk factors*				
Demographic and physiological status	Age Pregnancy/lactation	Usually defined by participant recall to questionnaires	Requires the knowledge of cultural norms	$
Socioeconomic characteristics	Income Educational attainment (incl. maternal education) Food insecurity Inequity, women’s empowerment	Usually defined by participant recall to questionnaires	Requires the knowledge of cultural norms	$
Lack of micronutrients and diversity in diet	Recent intake of animal source foods Recent intake of iron inhibitors (phytates, tannins) Recent consumption of foods fortified with iron, folate, and/or vitamin A Supplementation with iron, folate, and/or vitamin A	Usually defined by participant recall to questionnaires	Requires the knowledge of common dietary patterns among target population	$
Family planning practices	Onset of childbearing, parity, birth spacing	Usually defined by participant recall to questionnaires	Requires the knowledge of cultural norms	$
Health practices/health services	Antenatal care during previous pregnancy Emergency obstetric and neonatal care Modern contraceptives Malaria prevention practices, if applicable Deworming, if applicable	Usually defined by participant recall to questionnaires	Requires the knowledge of cultural norms and offered healthcare services	$
Water access, sanitation, and hygiene	WASH practices	Usually defined by participant recall to questionnaires	Requires the knowledge of local practices and programs	$
Social support programs	Poverty alleviation Income support programs	Defined at regional level or by participant recall to questionnaires	Requires the knowledge of local programs	$

Abbreviations: Hb, hemoglobin; RBC, red blood cell; SCH, schistosomiasis; STH, soil-transmitted helminthiasis; WASH, Water Access, Sanitation, and Hygiene.

a The cutoff depends on the laboratory method.

*Source*: Adapted with permission from USAID Advancing Nutrition.^16^
